# AGO2 protein: a key enzyme in the miRNA pathway as a novel biomarker in adrenocortical carcinoma

**DOI:** 10.1530/ERC-24-0061

**Published:** 2024-11-20

**Authors:** Anila Hashmi, Alexander Papachristos, Stan Sidhu, Gyorgy Hutvagner

**Affiliations:** 1 School of Biomedical Engineering, University of Technology Sydney, Sydney, New South Wales, Australia; 2 NSW Health Pathology, Sydney, New South Wales, Australia; 3 Endocrine Surgery Unit, Royal North Shore Hospital, Northern Sydney Local Health District, St Leonards, New South Wales, Australia; 4 Northern Clinical School, Sydney Medical School, Faculty of Medicine and Health, University of Sydney, Sydney, New South Wales, Australia; 5 Cancer Genetics Laboratory, Kolling Institute, Northern Sydney Local Health District, St. Leonards, New South Wales, Australia

**Keywords:** adrenocortical carcinoma, AGO2-protein, diagnostic and prognostic biomarkers, miRNA

## Abstract

Adrenocortical carcinoma (ACC) is a rare and aggressive malignancy characterized by diagnostic challenges, high recurrence rates, and poor prognosis. This study explored the role of miRNA processing genes in ACC and their potential role as diagnostic and prognostic biomarkers. We analyzed the mRNA expression levels of miRNA machinery components (DROSHA, DGCR8, XPO5, RAN, DICER, TARBP2, and AGO2) utilizing mRNA-Seq data from The Cancer Genome Atlas (TCGA) and The Genotype-Tissue Expression (GTEx) projects. Additionally, protein levels were quantified in tissue samples from the Kolling Institute of Medical Research’s tumor bank. Our results demonstrated that among all miRNA processing components, AGO2 exhibited significant overexpression in ACC compared to the normal adrenal cortex and benign adrenal adenoma (*P* < 0.001). Kaplan–Meier survival analysis indicated that higher AGO2 expression correlated with significantly worse overall survival in ACC patients (HR: 7.07, *P* < 0.001). Among 32 cancer types in TCGA, the prognostic significance of AGO2 was most prominent in ACC. This study is the first to report AGO2's potential as a diagnostic and prognostic biomarker in ACC, emphasizing its significance in ACC pathogenesis and potential application as a non-invasive liquid biopsy biomarker.

## Introduction

Adrenocortical carcinoma (ACC) is a rare and highly aggressive malignancy of the adrenal gland. Five-year survival rates vary based on disease stage at diagnosis, ranging from 60 to 80% for localized tumors to 0–28% for metastatic disease (Fassnacht *et al.* 2018). Currently, surgical resection remains the only curative therapeutic option. For unresectable diseases, systemic therapy is recommended by clinical practice guidelines; however, the efficacy of these treatments is limited, with objective response rates of less than 25% and significant side effects (Fassnacht *et al.* 2018, Turla *et al.* 2022). Even after curative resection, disease recurrence occurs in more than 60% of patients and poses a significant therapeutic challenge (Amini *et al.* 2016). To date, two comprehensive multi-omics studies have laid the foundation for understanding the molecular classification of ACC and its prognostic implications (Assié *et al.* 2014, Zheng *et al.* 2016). Zheng and colleagues classified the molecular signature of ACC into three groups based on a Cluster of Cluster (CoC) analysis of DNA copy number, DNA methylation, mRNA expression, and miRNA expression – COC1, COC2, and COC3 – each reflecting distinct prognostic outcomes, with COC1 showing the best prognosis and COC3 showing the worst. Moreover, despite advances in the genomic characterization of ACC (Assié *et al.* 2014, Zheng *et al.* 2016), there are currently no biomarkers that facilitate diagnosis, pathological prognostication, or monitoring for recurrent disease after curative resection (Fassnacht *et al.* 2016, Sinclair *et al.* 2020, Hazimeh *et al.* 2021).

miRNAs are small non-coding RNAs that regulate more than 60% of protein-coding genes by interacting with mRNA (Friedman *et al.* 2009). The differential expression of miRNAs between ACC and adrenal adenoma has recently emerged as a potential diagnostic and prognostic indicator. Specific miRNAs, such as the upregulation of miR-503, miR-210, miR-483-5p, and miR-483-3p and the downregulation of miR-195, miR-497, and miR-335, have been identified as potential markers for ACC (Decmann *et al.* 2020). However, the lack of significant differences in the expression of hsa-miR-483-3p and hsa-miR-483-5p between adrenal myelolipoma and ACC limits their clinical utility (Decmann *et al.* 2018). Furthermore, conflicting patterns of miRNA expression in ACC and adrenocortical adenoma (AA) have been reported (Özata *et al.* 2011, Koperski *et al.* 2017). These discrepancies highlight the complexity of miRNA regulation in ACC and the need for standardized quantification protocols and rigorous validation. Currently, the utility of miRNAs as biomarkers is limited by their low expressed concentrations, lack of standardized analytical methodologies, and lack of specificity to tumor types (Mytareli *et al.* 2021).

The miRNA biogenesis pathway consists of tightly regulated, interdependent steps involving key components such as DGCR8, drosha, exportin-5 (XPO5), RAN, dicer1, TARBP2, and AGO2, which are essential for miRNA maturation and function. This pathway has previously been extensively described (Moore & Blobel 1993, Bernstein *et al.* 2001, Hutvágner *et al.* 2001, Yi *et al.* 2003, Han *et al.* 2004, Lee *et al.* 2004, Chendrimada *et al.* 2005). In various cancers, such as clear cell renal carcinoma (Lee *et al.* 2019), ovarian carcinoma (Vaksman *et al.* 2012), leiomyosarcoma (Papachristou *et al.* 2012), and breast cancer (Yan *et al.* 2012), deregulation of miRNA-processing complexes has been observed, indicating their potential role in tumorigenesis. In this study, we evaluated the expression of miRNA biogenesis components in ACC. Among these components, argonaute 2 (AGO2) – a key regulator directing miRNAs to their target genes and modulating gene expression at the post-transcriptional level (Hutvagner & Simard 2008) – emerged as a candidate for further investigation. Through a comprehensive analysis of AGO2 and related miRNA genes, we aimed to explore their potential as novel diagnostic and prognostic biomarkers for ACC.

## Materials and methods

### RNA-seq data analysis for miRNA biogenesis genes in ACC

We obtained RNA-Seq data from two public repositories: The Cancer Genome Atlas (TCGA) for cancer samples and The Genotype-Tissue Expression (GTEx) project for normal tissue samples. Our bioinformatic analysis focused on the mRNA expression of core components in the miRNA biogenesis pathway, specifically AGO2, DGCR8, XPO5, RAN, DROSHA, DICER, and TARBP2, in ACC. Normalized RNA sequencing (RNA-Seq) data specific to miRNA biogenesis genes for normal adrenal cortical tissue were obtained from the Genotype-Tissue Expression (GTEx) project and from TCGA for ACC. The TNMplot bioinformatics web tool was used for data retrieval (Bartha & Győrffy 2021).

### Survival analysis

Survival analysis paired gene expression data and survival data from TCGA, using the Encyclopedia of RNA Interactomes (ENCORI) database (Li *et al.* 2014). Kaplan–Meier survival analysis was performed on the UCSC Xena platform (Goldman *et al.* 2020). To explore the specificity of the prognostic value of AGO2 expression for ACC, survival data for 32 different cancers, including clinicopathological data, were obtained from TCGA.

### Tumor samples

The study received ethics approval from the Northern Sydney Local Health District Human Research Ethics Committee (2020/ETH01931). Tissue samples, including ACC, benign adrenocortical adenoma (AA), and normal adrenal cortex (NAC) samples, were obtained from the Tumour Bank of the Kolling Institute of Medical Research. The Kolling Institute Tumour Bank Access Committee granted access to these samples (reference NETBMC #20–49). All participating patients provided informed consent for the use of their tissue samples and the collection of associated clinical data. At the time of adrenalectomy, tissue samples were immediately snap-frozen in liquid nitrogen and subsequently stored at −80°C. All ACC samples utilized in this study were histologically confirmed according to accepted diagnostic criteria (https://www.rcpa.edu.au/Library/Practising-Pathology/Structured-Pathology-Reporting-of-Cancer/Cancer-Protocols).

### Protein expression analysis

Snap-frozen tissue samples, including 15 NAC, 15 AA, and 15 ACC, were obtained from the Kolling Institute Tumour Bank. Tissue homogenates were prepared by washing the tissue with pre-cooled phosphate-buffered saline (PBS) buffer (0.01M, pH = 7.4). The tissue samples were then homogenized in Lysing Matrix A tubes (MP Biomedicals, Australia). Homogenization was performed using a FastPrep-24™5G (MP Biomedicals) bead-beating grinder and lysis system according to the manufacturer's guidelines. Protein expression levels of miRNA biogenesis genes were measured using Human Protein ELISA Kits according to the manufacturer’s instructions and included AGO2, DGCR8, DROSHA, RAN, XPO5 (Abebio-Co. Ltd.), and TARBP2 and DICER1 (Fine Biotech Co., Ltd.). Protein concentrations were measured by comparing the optical density to standard controls using a microplate reader (TECAN Spark absorbance reader).

### Analysis of clinicopathological parameters and AGO2 expression

We examined the relationships between AGO2 expression and key clinicopathological parameters, including age, sex, overall survival status, Weiss score, adrenal hormone excess, and tumor stage. mRNA expression data for AGO2 and clinicopathological information were obtained from TCGA for 79 ACC patients (Cerami *et al.* 2012). Independent protein expression data were obtained from a cohort of 15 patients via the Kolling Tumour Bank.

### miRNA-AGO2 correlation analysis

We identified the top miRNAs highly expressed in TCGA-ACC patient clusters that are associated with distinct prognostic outcomes. The expression levels of these selected miRNAs were then correlated with AGO2 mRNA expression within the same patient cohort. For the identification of highly expressed miRNAs, we utilized supplementary data (see section on supplementary materials given at the end of this article) from the TCGA-ACC project (Zheng *et al.* 2016) and assessed the correlation of these identified miRNAs with AGO2 mRNA expression levels using the ENCORI platform (The Encyclopedia of RNA Interactomes) (Li *et al.* 2014). Our objective through this approach was to examine the correlation between the selected miRNAs and AGO2 expression, contributing to our understanding of AGO2's role in ACC pathogenesis.

### Statistical analysis

Statistical analysis was performed using GraphPad Prism, version 9 (GraphPad Software). For gene expression data analysis, two-way ANOVA was used to compare the expression levels between groups. The log-rank test was used to compare survival outcomes between groups; for both gene expression and gene survival analysis, a *P*-value of <0.05 was considered statistically significant. To explore the correlation between gene expression and tumor staging in ACC, one-way ANOVA was utilized with a *P*-value threshold of <0.05. ELISA absorbance levels were interpreted based on the construction of a standard curve in Microsoft Excel (Version 2306 Build 16.0.16529.20166) and Curve Expert Basic (V.1.4-USA), with protein levels compared using one-way ANOVA and a *P*-value threshold of <0.05. The receiver operating characteristic (ROC) curve was used to determine the optimal cut-off point for AGO2 protein levels, balancing sensitivity and specificity in the diagnosis of ACC.

Additionally, DataTab (https://datatab.net/) was utilized to perform statistical analyses of AGO2 mRNA expression in the TCGA-ACC cohort and AGO2 protein concentration in the collected ACC cohort. Independent *t*-tests were applied to assess the significance of correlations between AGO2 expression levels and various clinicopathological parameters, including age at diagnosis, sex, tumor stage, Weiss score, and overall survival outcomes. A significance threshold was established at a *P*-value of <0.05.

## Results

### Differential expression of miRNA biogenesis genes in ACC and NAC

**Figure 1:**
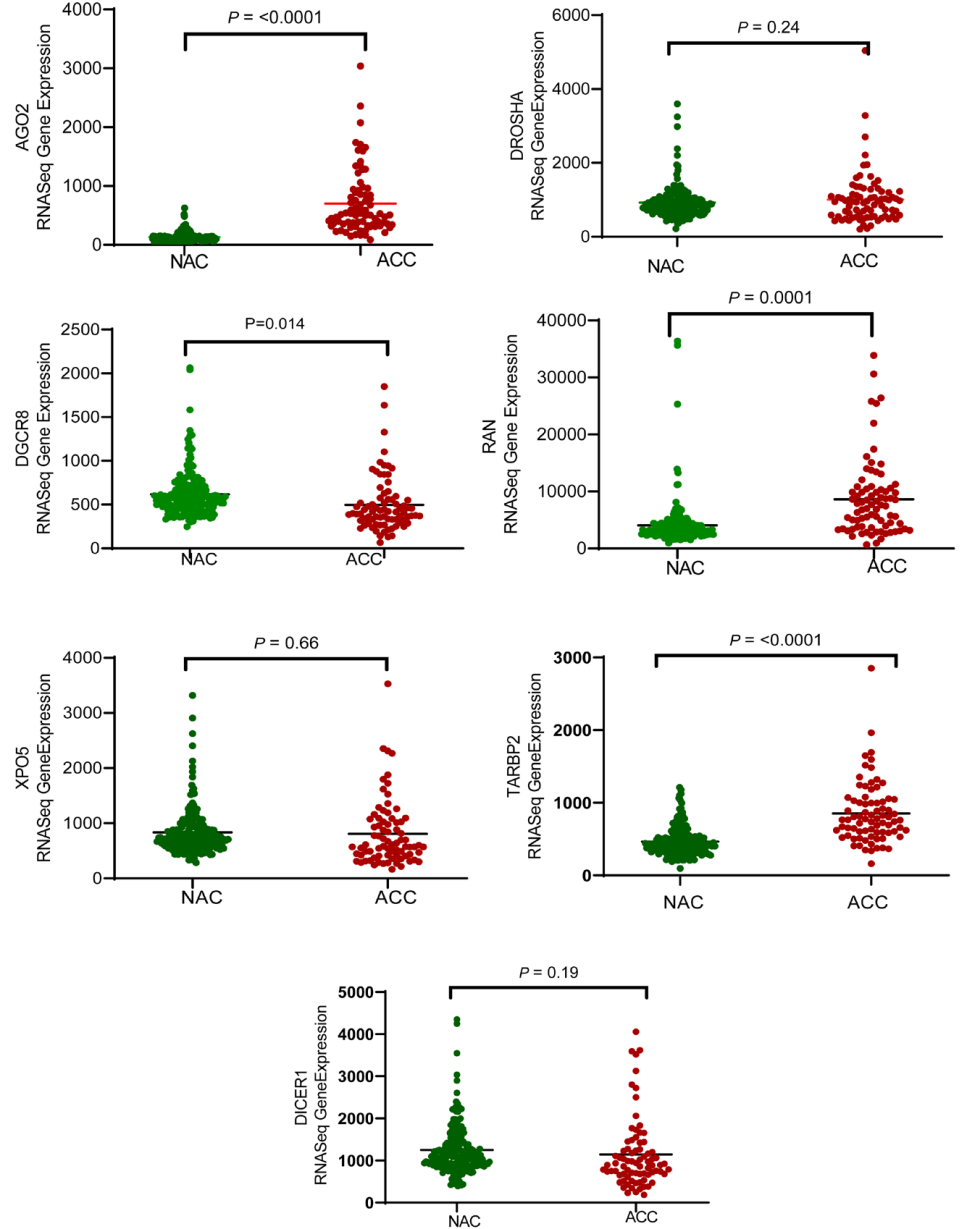
mRNA expression analysis of genes related to miRNA biogenesis in adrenocortical carcinoma (ACC) and normal adrenal cortex (NAC) tissue samples. The expression levels of miRNA biogenesis genes (AGO2, DROSHA, DGCR8, XPO5, RAN, TARBP2, and DICER1) were compared in ACC and normal adrenal cortex tissue samples using RNA-Seq data from the TCGA and GTEX datasets. Among these genes, AGO2 showed significantly higher expression in ACC samples than in normal samples (*P* < 0.001), whereas minimal or no expression of AGO2 was detected in normal samples. A full-color version of this figure is available at https://doi.org/10.1530/ERC-24-0061.

According to the RNA-Seq data from the GTEx project and TCGA, AGO2, RAN, and TARBP2 were significantly upregulated in ACC samples compared to NAC samples (*P* ≤ 0.001). Conversely, DGCR8 expression was slightly higher in the NAC than in ACC (*P* = 0.014). No statistically significant differences were observed in the expression levels of DROSHA (*P* = 0.24), DICER1 (*P* = 0.19), and XPO5 (*P* = 0.66) (Fig. 1).

### Among all miRNA biogenesis genes, AGO2 is the strongest prognostic indicator in ACC

**Figure 2:**
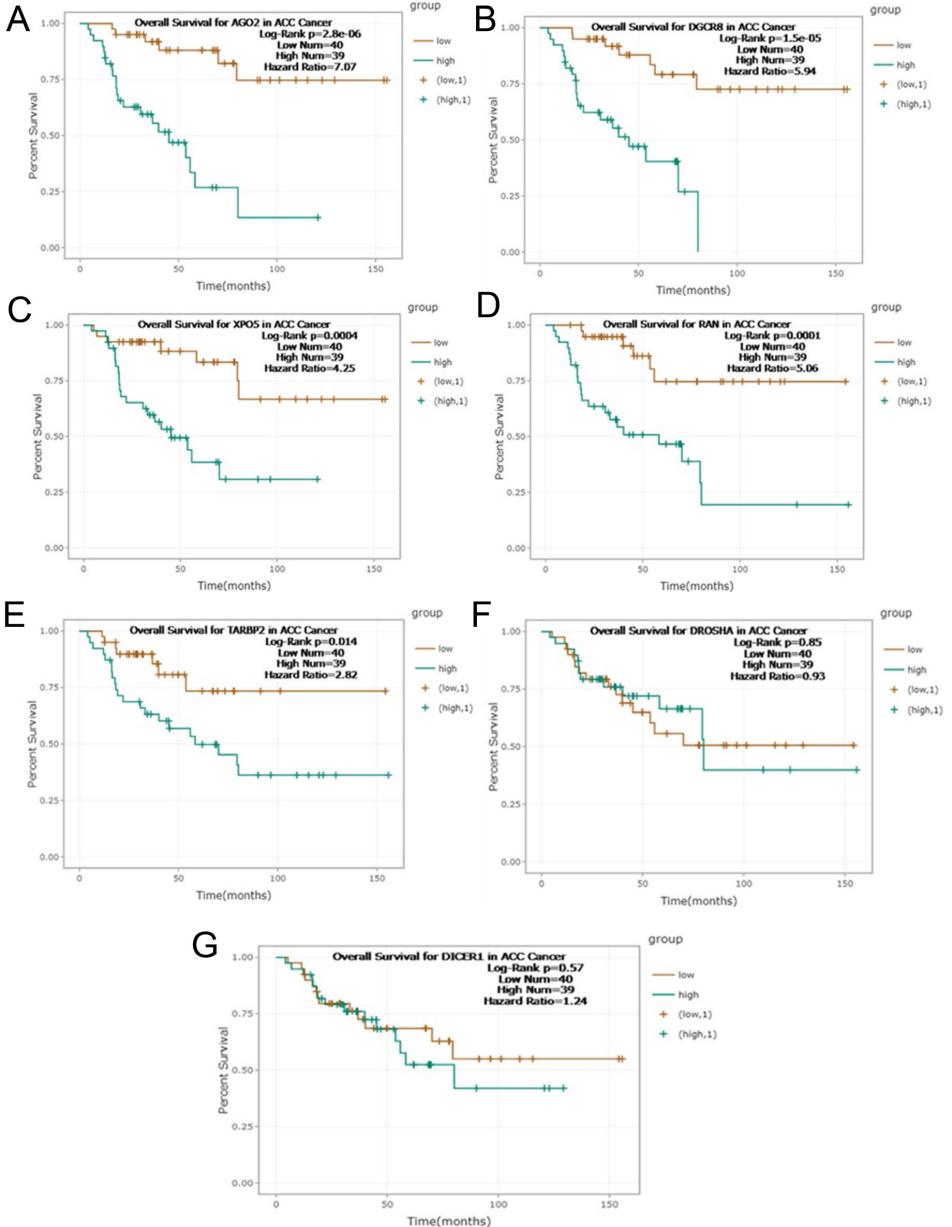
Association between miRNA biogenesis gene expression and survival rates in adrenocortical carcinoma (ACC) patients. Gene survival analysis of TCGA RNA-seq data was performed to explore overall survival rates in 79 ACC patients with adrenocortical carcinoma according to high (green) or low (brown) gene expression levels. The analysis revealed a poor prognosis associated with high expression levels of AGO2, DGCR8, XPO5, and RAN, with log-rank *P* <0.001. TARBP2 showed a weaker prognostic association (log-rank *P* = 0.014). DROSHA and DICER did not exhibit significant prognostic correlations, with log-rank *P* = 0.85 and *P* = 0.57, respectively. Among the genes involved in the miRNA biogenesis pathway, AGO2 emerged as the strongest prognostic indicator in ACC, exhibiting a hazard ratio (HR) of 7.07 and a log-rank test *P*-value of 2.8e-06.

**Figure 3:**
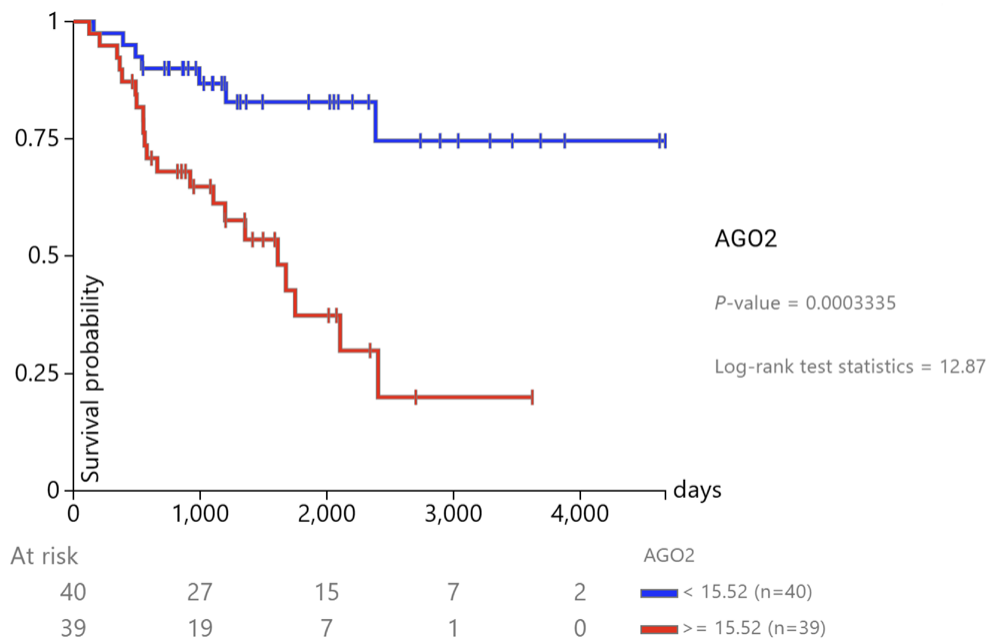
Kaplan‒Meier gene expression analysis of AGO2-ACC-TCGA. Kaplan‒Meier curves comparing survival between ACC patients with low (< 15.52, blue) and high (≥ 15.52, red) AGO2 expression in the TCGA cohort. The difference in survival was statistically significant (*P* = 0.0003335, log-rank test statistic = 12.87), indicating a prognostic impact of AGO2 expression on patient outcome.

To assess the prognostic value of miRNA biogenesis genes in ACC, we utilized RNA-seq data from TCGA. For the survival analysis, cancer samples were divided into two groups based on the median expression of each gene, as per the guidelines provided by ENCORI. Among the genes involved in the miRNA biogenesis pathway, AGO2 emerged as the strongest prognostic indicator in ACC, exhibiting a hazard ratio (HR) of 7.07 and a log-rank test *P*-value of 2.8e-06 (Fig. 2). The Kaplan–Meier analysis further validated the strong association of AGO2 with poor prognosis in ACC patients (Fig. 3). Other genes, such as DGCR8, XPO5, and RAN, also demonstrated prognostic potential, but to a lesser extent, with HRs of 5.9 (*P* < 0.0001), 4.25 (*P* = 0.0004), and 5.06 (*P* = 0.0001), respectively. TARBP2 showed a weaker prognostic association, with an HR of 2.82 (*P* = 0.014). On the other hand, DROSHA and DICER did not exhibit significant prognostic correlations, with HRs of 0.93 (*P* = 0.85) and 1.24 (*P* = 0.57), respectively.

### The prognostic significance of the AGO2 gene in ACC is distinct from that in other cancers

The prognostic correlation of AGO2 gene expression was strongest in ACC (HR: 7.07, *P* = 2.8e-06) compared to the 31 other TCGA cancer types studied. Although AGO2 gene expression held prognostic relevance in cholangiocarcinoma (HR: 0.38, *P* = 0.044), renal cell carcinoma (HR: 2.15, *P* = 0.016), mesothelioma (HR: 2.36, *P* = 0.00053), sarcoma (HR: 1.71, *P* = 0.0092), and endometrial carcinoma (HR: 1.83, *P* = 0.0052), in none of these other cancer types did AGO2 demonstrate such a significant prognostic impact as in ACC (Supplementary Table 1).

### High AGO2 protein expression in ACC compared to benign adrenal cortex and NAC

**Figure 4:**
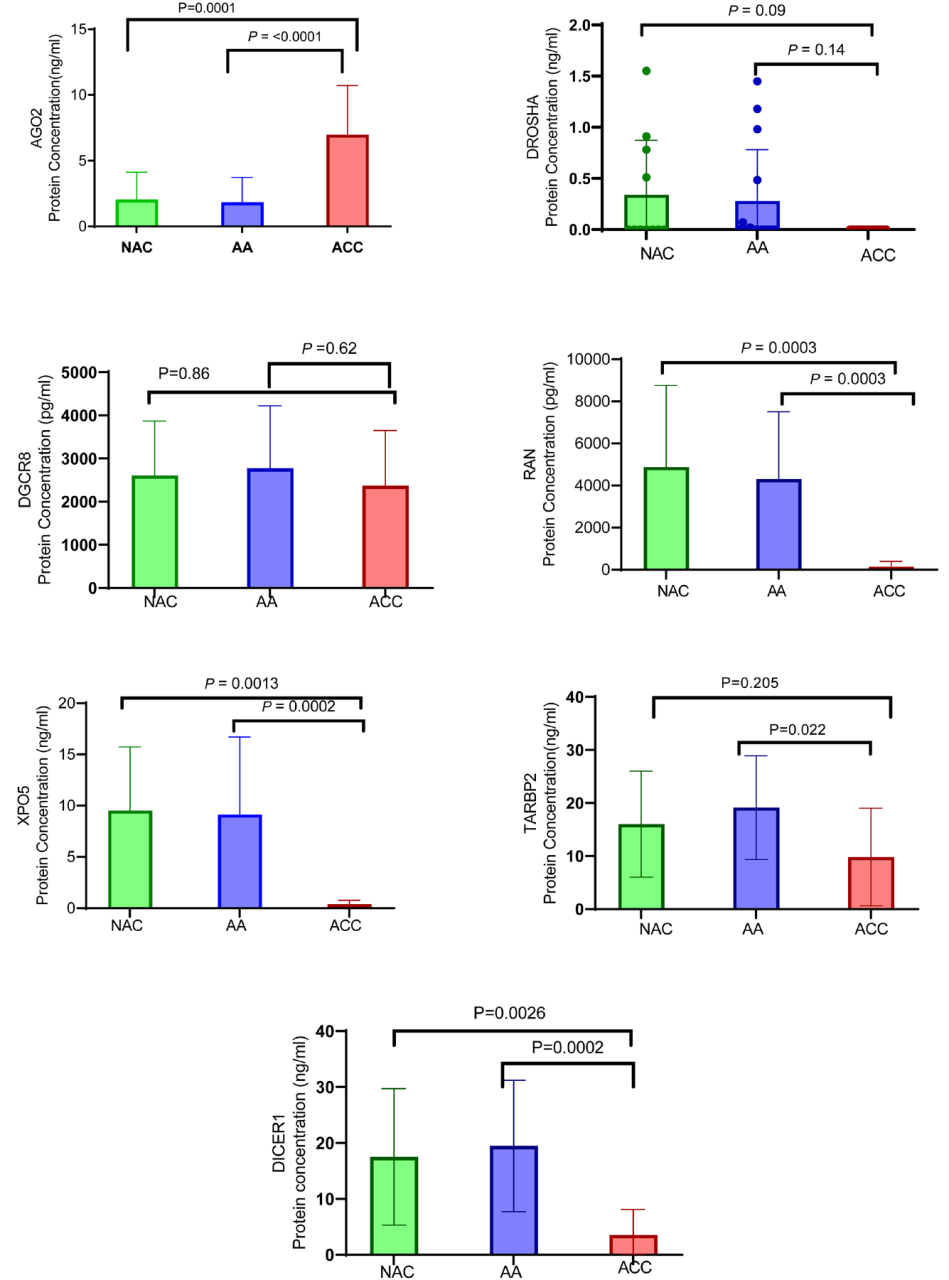
Protein expression analysis of miRNA biogenesis components in adrenocortical carcinoma (ACC), normal, and benign tissue samples. The protein expression levels of AGO2, DROSHA, DGCR8, XPO5, RAN, TARBP2, and DICER1 in normal, benign, and adrenocortical cancer tissue homogenate samples were measured using ELISA. The results revealed that XPO5, RAN, TARBP2, and DICER1 protein expression was downregulated in cancer samples compared to both normal and benign samples, suggesting a potential role for these proteins in cancer development through post-translational modification. In contrast, AGO2 protein expression was significantly higher in cancer samples than in both normal and benign samples. These findings highlight AGO2 as a strong candidate potential diagnostic biomarker for adrenocortical carcinoma among all the miRNA biogenesis factors analyzed. A full-colour version of this figure is available at https://doi.org/10.1530/ERC-24-0061.

AGO2 protein concentration was significantly higher in ACC than in adrenal adenoma or NAC (*P* < 0.0001). Furthermore, there was no significant difference in AGO2 protein expression between normal and benign tumors (Fig. 4). In contrast, XPO5, RAN, and DICER1 protein expression levels were significantly lower in ACC tissue homogenate samples compared to the non-malignant tissue samples (*P* < 0.001). No statistically significant differences were observed in the protein expression levels of DROSHA, DGCR8, or TARBP2 between the malignant and non-malignant groups.

#### ROC analysis and specific cut-off point determination

**Figure 5:**
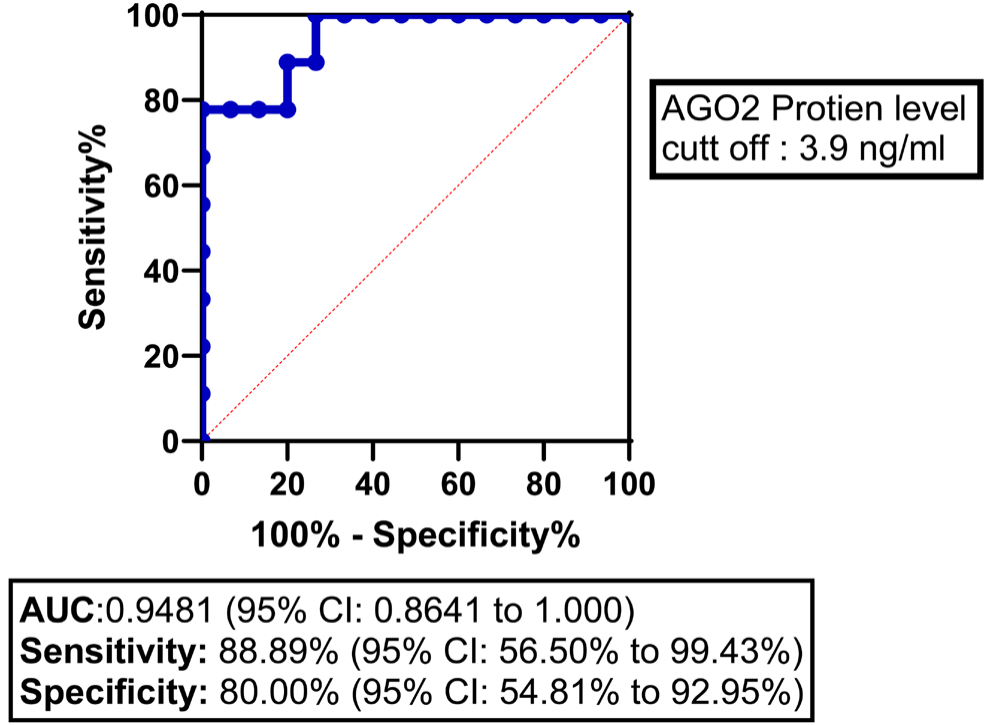
Receiver operating characteristic (ROC) curve for AGO2 protein expression in adrenocortical carcinoma (ACC) patients. The ROC curve illustrates the diagnostic ability of AGO2 protein expression to differentiate between ACC and non-malignant samples. The area under the curve (AUC) was 0.9481 (95% CI: 0.8641 to 1.000), indicating high diagnostic accuracy. A cut-off value of >3.9 for AGO2 protein expression yielded a sensitivity of 88.89% (95% CI: 56.50% to 99.43%) and a specificity of 80.00% (95% CI: 54.81% to 92.95%). The diagonal dashed line represents the line of no discrimination (AUC = 0.5).

To explore the appropriate diagnostic threshold for determining the level of the AGO2 protein in ACC compared to non-malignant tissue, we performed ROC curve analysis. The area under the curve (AUC) was 0.95 (95% CI: 0.86–1.00), indicating high diagnostic accuracy. Using a cut-off point of >3.9 ng/mL for AGO2 protein expression, a sensitivity of 89% (95% CI: 57–99%) and a specificity of 80% (95% CI: 55–93%) were achieved (Fig. 5).

## Associations between clinicopathological characteristics and AGO2 expression

The prognostic potential of AGO2 in ACC was further explored by correlating clinicopathological characteristics with AGO2 mRNA expression in the TCGA-ACC cohort (Cerami *et al.* 2012) and with the concentration of the AGO2 protein in a cohort from the Kolling Tumour Bank. The associations between clinicopathological characteristics and AGO2 mRNA expression and protein concentration are shown in Supplementary Table 2. Our findings indicate that neither AGO2 gene expression (*P* = 0.672) nor protein concentration (*P* = 0.833) significantly correlates with age at diagnosis, suggesting that their prognostic relevance is not influenced by patient age. Similarly, sex did not significantly affect AGO2 levels in either analysis (gene expression, *P* = 0.254; protein concentration, *P* = 0.484). Notably, overall survival status was significantly associated with AGO2 levels; patients who were deceased exhibited higher levels of AGO2, both at the gene (*P* < 0.001) and protein levels (*P* = 0.009). The Weiss score, which reflects tumor aggressiveness, further confirmed this finding, with higher scores correlating with elevated AGO2 expression (gene *P* = 0.003, protein *P* = 0.008). Additionally, AGO2 expression levels varied significantly with pathological stage, with advanced-stage tumors (III–IV) showing increased levels compared to early-stage tumors (I–II) (gene *P* = 0.011, protein *P* = 0.004).

When correlating AGO2 mRNA expression within the TCGA-ACC dataset, which categorizes ACC into three distinct molecular subtypes (CoC1, CoC2, and CoC3), we observed notable prognostic disparities. Specifically, in the COC1 group, with a disease progression rate of 7%, the mean AGO2 mRNA expression was −0.16 ± 0.91 (log2). In the COC2 group, with a disease progression rate of 56%, the mean expression was −0.18 ± 1.14 (log2). Most notably, the COC3 group, characterized by the most adverse outcomes and a high disease progression rate of 96%, exhibited significantly higher levels of AGO2 expression (mean 0.31 ± 1.01 log2) compared to the COC1 group (*P*-value = 0.036). This finding underscores the potential role of AGO2 as a prognostic indicator in ACC.

### AGO2 expression in relation to overall survival and hormone production in ACC

**Figure 6:**
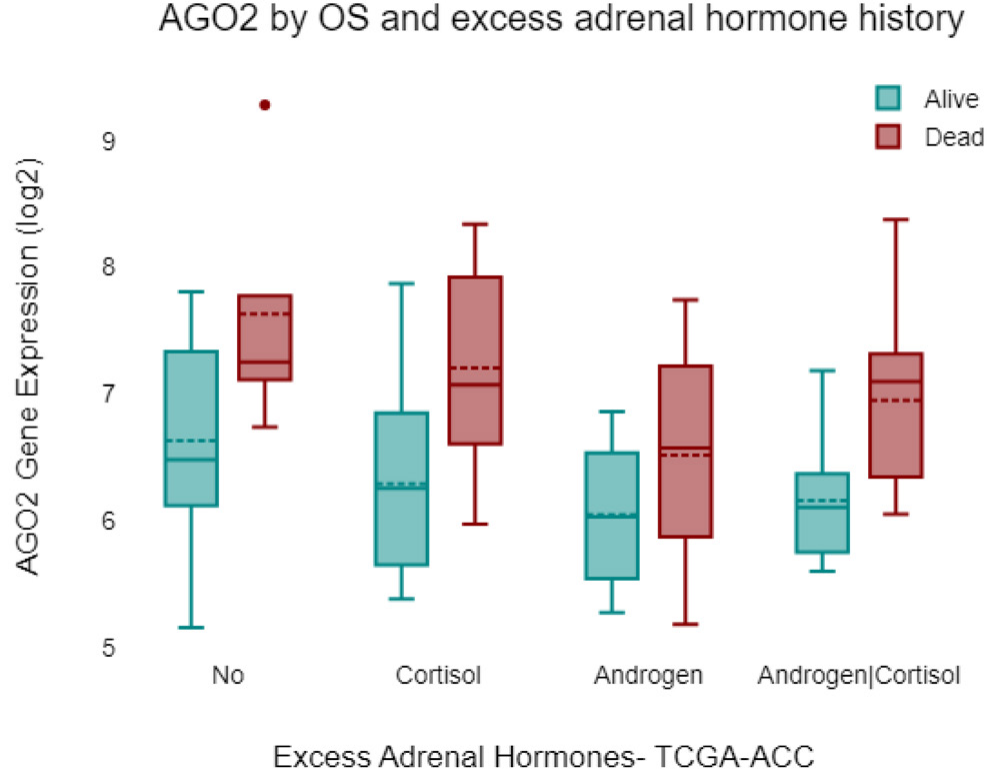
Association of AGO2 expression with overall survival (OS) and excess adrenal hormone history in ACC. AGO2 gene expression (log2-transformed) is shown across different hormone production statuses in ACC patients from the TCGA-ACC cohort: No excess hormone production, Cortisol, Androgen, and Androgen|Cortisol. Across all groups, deceased patients (red) have higher AGO2 expression compared to those alive, indicating that elevated AGO2 is associated with poor survival outcomes. Notably, even patients with no excess hormone production show a positive correlation between AGO2 expression and survival, highlighting AGO2 as a potentially better prognostic marker than hormone production status alone.

AGO2 gene expression (log2-transformed) was analyzed in relation to overall survival (OS) and excess adrenal hormone status in TCGA-ACC patients (Fig. 6). The data were categorized into groups based on hormone secretion: No excess hormone production, Cortisol, Androgen, and Androgen|Cortisol. Elevated AGO2 expression was observed in deceased patients across all hormone statuses. Notably, patients with no excess hormone production also demonstrated higher AGO2 expression in the deceased cohort, suggesting that AGO2 expression is associated with poor prognosis independent of hormone production. Furthermore, deceased patients generally exhibited higher AGO2 expression compared to those alive within each hormone category, indicating the potential utility of AGO2 as a prognostic biomarker in ACC.

## Differential AGO2-miRNA expression correlated with prognostic disparities in ACC clusters

We conducted a correlation analysis to explore the relationship between AGO2 mRNA expression and miRNA expression profiles within TCGA-ACC patient clusters, which are distinguished by their prognostic outcomes. Notably, within the COC3 cluster – identified as having the worst prognosis and the highest rate of disease progression – a significant positive correlation was observed between AGO2 expression and the four most highly expressed miRNAs: hsa-miR-196a-5p (*r* = 0.351, *P*-value = 1.54e-03), hsa-miR-182-5p (*r* = 0.357, *P*-value = 1.25e-03), hsa-miR-139-3p (*r* = 0.324, *P*-value = 3.56e-03), and hsa-miR-183-5p (*r* = 0.397, *P*-value = 2.90e-04) (Supplementary Figure 1A). This positive correlation suggests a possible role of these miRNAs in conjunction with AGO2 in driving the aggressive nature of ACC within this patient group.

Conversely, the COC1 group, characterized by a more favorable prognosis, demonstrated an inverse correlation between AGO2 and miRNAs from the cluster Xq27.3 (Yoshida *et al.* 2021). Specifically, the miRNAs hsa-miR-513c-5p (*r* = −0.401, *P*-value = 2.51e-04), hsa-miR-506-3p (*r* = −0.393, *P*-value = 3.47e-04), hsa-miR-514a-3p (*r* = −0.389, *P*-value = 3.89e-04), and hsa-miR-513a-5p (*r* = −0.442, *P*-value = 4.52e-05) all exhibited a negative correlation with AGO2 expression. This inverse relationship may indicate the potential of these miRNAs, in conjunction with lower AGO2 expression, to mediate a less aggressive disease phenotype in COC1 patients (Supplementary Figure 1B).

## Discussion

In this study, we demonstrated the potential role of AGO2 as a diagnostic and prognostic marker in ACC. AGO2 is a key regulator of miRNA function and maturation (Connerty *et al.* 2015), with variable expression across cancer types (Ye *et al.* 2015). Our analysis revealed a positive correlation between AGO2 expression and adverse clinical outcomes in ACC, including poorer survival, higher Weiss scores, and advanced tumor stages, emphasizing its potential as a biomarker. When evaluating AGO2 expression across TCGA-ACC clusters (COC1, COC2, and COC3) (Zheng *et al.* 2016), the COC3 group, which has the worst prognosis, exhibited significantly higher AGO2 levels compared to COC1 and COC2, indicating a potential role of AGO2 in the pathogenesis of aggressive ACC. Although other proteins like TARBP2, RAN, and XPO5 showed expression differences, they lacked the concordance and prognostic significance of AGO2.

Previous studies that have examined the prognostic impact of miRNA biogenesis proteins have reported conflicting results. For example, Caramuta (Caramuta *et al.* 2013) reported the upregulation of TARBP2 mRNA levels in ACC patients, whereas de Sousa (Sousa *et al.* 2015) reported no difference in TARBP2 gene or protein (TRBP) expression between AAs and ACC. In our study, although TARBP2 and RAN gene expression was significantly increased in ACC, a corresponding increase in protein expression was not detected. Conversely, the gene expression of DROSHA, XPO5, and DICER did not differ between ACC and NAC; however, the protein expression levels were significantly lower in ACC. These discrepancies not only highlight the complexity of post-transcriptional and post-translational regulatory mechanisms on protein expression levels in ACC but also highlight the inherent challenges in comparing different methodological quantitative approaches.

Our investigation revealed a notable positive correlation between the expression of AGO2 and that of the four most highly expressed miRNAs in the COC3 cluster (hsa-miR-196a-5p, hsa-miR-182-5p, hsa-miR-139-3p, and hsa-miR-183-5p), which are associated with poor prognosis. In contrast, the COC1 cluster, which is associated with a more favorable prognosis, exhibited an inverse correlation with AGO2 expression (Zheng *et al.* 2016). Considering the extensive progression rate of COC3, AGO2 merits further investigation to explore its role in ACC pathogenesis and its potential role as a diagnostic and prognostic biomarker.

In progressing toward clinical translation, several considerations must be addressed. Establishing the cut-off point for AGO2 protein expression is important. Furthermore, comparing AGO2 protein levels in tissue samples and blood samples may facilitate further investigation into its potential application as a liquid biopsy. Similarly, further investigation into the quantitative significance of AGO2 protein levels in early-stage tumors may be useful in guiding adjuvant treatment and follow-up protocols.

## Limitations

While we validated the elevated mRNA expression of AGO2 in the TCGA and GTEx cohorts, relevant cut-off values for AGO2 protein expression in ACC require additional clinical trials and validation in larger cohorts.

## Conclusion

This study is the first to identify Argonaute 2 (AGO2), a key regulator of miRNA function, as a potential diagnostic and prognostic biomarker in ACC. AGO2 is upregulated in ACC compared to adrenal adenoma and the NAC. This upregulation is evident at both the gene and protein levels, distinguishing AGO2 from other miRNA biogenesis proteins evaluated in this study. Compared to 31 other cancers in the TCGA dataset, the degree and significance of the prognostic impact of AGO2 expression are unique to ACC. The strong association between AGO2 expression and clinicopathological outcomes underlines its potential role in ACC progression. This study lays the groundwork for future research, especially in exploring the feasibility of AGO2 as a liquid biopsy biomarker – a promising direction that could revolutionize non-invasive cancer diagnostics and prognostication in ACC.

## Supplementary Materials

Supplementary Figure 1A:Co-expression of AGO2 with prognostically significant miRNAs in ACC The top four miRNAs highly expressed in the COC3 group-TCGA-ACC, which is associated with poor prognosis (hsa-miR-183-5p, hsa-miR-139-3p, hsa-miR-182-5p, and hsa-miR-196a-5p), demonstrated a positive correlation with AGO2 expression.

Supplementary Table 1: Pan-Cancer AGO2 expression and survival analysis in TCGA cohorts. This table summarizes the hazard ratios (HR) for AGO2 expression across 32 TCGA cancer types, highlighting its prognostic significance, particularly in ACC with an HR of 7.07 (p value 2.80E-06)

Supplementary Table 2: Comparative Analysis of AGO2 mRNA and Protein Levels Against Clinicopathological Parameters and Their Prognostic Significance in TCGA-ACC and an Independent ACC Cohort.

## Declaration of interest

The authors declare that there is no conflict of interest that could be perceived as prejudicing the impartiality of the study reported.

## Funding

This project was funded by Professor Stanley Sidhu of the Cancer Genetics Laboratory at Kolling Institute of Medical Research (NSLHD Cost Code CC 304618).

## Ethics approval and consent to participate

The study received ethics approval from the Northern Sydney Local Health District Human Research Ethics Committee (2020/ETH01931). Tissue samples, including adrenocortical carcinoma (ACC), benign adrenocortical adenoma (AA), and normal adrenal cortex (NAC) samples, were obtained from the Tumour Bank of the Kolling Institute of Medical Research. The Kolling Institute Tumour Bank Access Committee granted access to these samples (reference NETBMC #20-49). All participating patients provided informed consent for the use of their tissue samples and the collection of associated clinical data.

## Author contribution statement

AH designed the study, performed the experiments, interpreted the results, and drafted the manuscript. GH and SS supervised the study design. AH, GH, SS, and AP contributed to the scientific discussion, manuscript editing, and the finalization of the manuscript. All the authors have read and agreed to the published version of the manuscript.

## References

[bib1] Amini N Margonis GA Kim Y Tran TB Postlewait LM Maithel SK Wang TS Evans DB Hatzaras I Shenoy R, *et al.* 2016 Curative resection of adrenocortical carcinoma: rates and patterns of postoperative recurrence. Annals of Surgical Oncology 23 126–133. (10.1245/s10434-015-4810-y) 26282907 PMC4962540

[bib2] Assié G Letouzé E Fassnacht M Jouinot A Luscap W Barreau O Omeiri H Rodriguez S Perlemoine K René-Corail F, *et al.* 2014 Integrated genomic characterization of adrenocortical carcinoma. Nature Genetics 46 607–612. (10.1038/ng.2953) 24747642

[bib3] Bartha Á & Győrffy B 2021 TNMplot.com: a web tool for the comparison of gene expression in normal, tumor and metastatic tissues. International Journal of Molecular Sciences 22 2622. (10.3390/ijms22052622) 33807717 PMC7961455

[bib4] Bernstein E Caudy AA Hammond SM & Hannon GJ 2001 Role for a bidentate ribonuclease in the initiation step of RNA interference. Nature 409 363–366. (10.1038/35053110) 11201747

[bib5] Caramuta S Lee L Özata DM Akçakaya P Xie H Höög A Zedenius J Bäckdahl M Larsson C & Lui W-O 2013 Clinical and functional impact of TARBP2 over-expression in adrenocortical carcinoma. Endocrine-Related Cancer 20 551–564. (10.1530/ERC-13-0098) 23671264 PMC3709642

[bib6] Cerami E Gao J Dogrusoz U Gross BE Sumer SO Aksoy BA Jacobsen A Byrne CJ Heuer ML Larsson E, *et al.* 2012 The cBio cancer genomics portal: an open platform for exploring multidimensional cancer genomics data. Cancer Discovery 2 401–404. (10.1158/2159-8290.CD-12-0095) 22588877 PMC3956037

[bib7] Chendrimada TP Gregory RI Kumaraswamy E Norman J Cooch N Nishikura K & Shiekhattar R 2005 TRBP recruits the Dicer complex to Ago2 for microRNA processing and gene silencing. Nature 436 740–744. (10.1038/nature03868) 15973356 PMC2944926

[bib8] Connerty P Ahadi A & Hutvagner G 2015 RNA binding proteins in the miRNA pathway. International Journal of Molecular Sciences 17 31. (10.3390/ijms17010031) 26712751 PMC4730277

[bib9] Decmann A Perge P Nyírő G Darvasi O Likó I Borka K Micsik T Tóth Z Bancos I Pezzani R, *et al.* 2018 MicroRNA expression profiling in adrenal myelolipoma. Journal of Clinical Endocrinology and Metabolism 103 3522–3530. (10.1210/jc.2018-00817) 29982598

[bib10] Decmann A Perge P Turai PI Patócs A & Igaz P 2020 Non-coding RNAs in adrenocortical cancer: from pathogenesis to diagnosis. Cancers 12 461. (10.3390/cancers12020461) 32079166 PMC7072220

[bib11] Fassnacht M Arlt W Bancos I Dralle H Newell-Price J Sahdev A Tabarin A Terzolo M Tsagarakis S & Dekkers OM 2016 Management of adrenal incidentalomas: European society of endocrinology clinical practice guideline in collaboration with the European network for the study of adrenal tumors. European Journal of Endocrinology 175 G1–G34. (10.1530/EJE-16-0467) 27390021

[bib12] Fassnacht M Dekkers OM Else T Baudin E Berruti A de Krijger R Haak HR Mihai R Assie G & Terzolo M 2018 European society of endocrinology clinical practice guidelines on the management of adrenocortical carcinoma in adults, in collaboration with the European network for the study of adrenal tumors. European Journal of Endocrinology 179 G1–G46. (10.1530/EJE-18-0608) 30299884

[bib13] Friedman RC Farh KK-H Burge CB & Bartel DP 2009 Most mammalian mRNAs are conserved targets of microRNAs. Genome Research 19 92–105. (10.1101/gr.082701.108) 18955434 PMC2612969

[bib14] Goldman MJ Craft B Hastie M Repečka K McDade F Kamath A Banerjee A Luo Y Rogers D Brooks AN, *et al.* 2020 Visualizing and interpreting cancer genomics data via the Xena platform. Nature Biotechnology 38 675–678. (10.1038/s41587-020-0546-8) PMC7386072 32444850

[bib15] Han J Lee Y Yeom K-H Kim Y-K Jin H & Kim VN 2004 The Drosha-DGCR8 complex in primary microRNA processing. Genes and Development 18 3016–3027. (10.1101/gad.1262504) 15574589 PMC535913

[bib16] Hazimeh Y Sigel C Carie C Leinung M & Khalaf Z 2021 Adrenocortical carcinoma: a case of missed diagnosis. Cureus 13 e14235. (10.7759/cureus.14235) 33948420 PMC8087872

[bib17] Hutvágner G McLachlan J Pasquinelli AE Bálint E Tuschl T & Zamore PD 2001 A cellular function for the RNA-Interference enzyme Dicer in the maturation of the *let-7* small temporal RNA. Science 293 834–838. (10.1126/science.1062961) 11452083

[bib18] Hutvagner G & Simard MJ 2008 Argonaute proteins: key players in RNA silencing. Nature Reviews. Molecular Cell Biology 9 22–32. (10.1038/nrm2321) 18073770

[bib19] Koperski Ł Kotlarek M Świerniak M Kolanowska M Kubiak A Górnicka B Jażdżewski K & Wójcicka A 2017 Next-generation sequencing reveals microRNA markers of adrenocortical tumors malignancy. Oncotarget 8 49191–49200. (10.18632/oncotarget.16788) 28423361 PMC5564760

[bib20] Lee Y Kim M Han J Yeom K-H Lee S Baek SH & Kim VN 2004 MicroRNA genes are transcribed by RNA polymerase II. EMBO Journal 23 4051–4060. (10.1038/sj.emboj.7600385) 15372072 PMC524334

[bib21] Lee SS Min H Ha JY Kim BH Choi MS & Kim S 2019 Dysregulation of the miRNA biogenesis components DICER1, DROSHA, DGCR8 and AGO2 in clear cell renal cell carcinoma in both a Korean cohort and the cancer genome atlas kidney clear cell carcinoma cohort. Oncology Letters 18 4337–4345. (10.3892/ol.2019.10759) 31516620 PMC6732956

[bib22] Li J-H Liu S Zhou H Qu L-H & Yang J-H 2014 starBase v2.0: decoding miRNA-ceRNA, miRNA-ncRNA and protein–RNA interaction networks from large-scale CLIP-Seq data. Nucleic Acids Research 42 D92–D97. (10.1093/nar/gkt1248) 24297251 PMC3964941

[bib23] Moore MS & Blobel G 1993 The GTP-binding protein Ran/TC4 is required for protein import into the nucleus. Nature 365 661–663. (10.1038/365661a0) 8413630

[bib24] Mytareli C Delivanis DA Athanassouli F Kalotychou V Mantzourani M Kassi E & Angelousi A 2021 The diagnostic, prognostic and therapeutic role of miRNAs in adrenocortical carcinoma: a systematic review. Biomedicines 9 1501. (10.3390/biomedicines9111501) 34829730 PMC8614733

[bib25] Özata DM Caramuta S Velázquez-Fernández D Akçakaya P Xie H Höög A Zedenius J Bäckdahl M Larsson C & Lui W-O 2011 The role of microRNA deregulation in the pathogenesis of adrenocortical carcinoma. Endocrine-Related Cancer 18 643–655. (10.1530/ERC-11-0082) 21859927 PMC3201061

[bib26] Papachristou DJ Sklirou E Corradi D Grassani C Kontogeorgakos V & Rao UNM 2012 Immunohistochemical analysis of the endoribonucleases Drosha, Dicer and Ago2 in smooth muscle tumours of soft tissues. Histopathology 60 E28–E36. (10.1111/j.1365-2559.2012.04192.x) 22394132

[bib27] Sinclair TJ Gillis A Alobuia WM Wild H & Kebebew E 2020 Surgery for adrenocortical carcinoma: when and how? Best Practice and Research. Clinical Endocrinology and Metabolism 34 101408. (10.1016/j.beem.2020.101408) 32265101

[bib28] Sousa de GRV Ribeiro TC Faria AM Mariani BMP Lerario AM Zerbini MCN Soares IC Wakamatsu A Alves VAF Mendonca BB, *et al.* 2015 Low DICER1 expression is associated with poor clinical outcome in adrenocortical carcinoma. Oncotarget 6 22724–22733. (10.18632/oncotarget.4261) 26087193 PMC4673194

[bib29] Turla A Laganà M Grisanti S Abate A Ferrari VD Cremaschi V Sigala S Consoli F Cosentini D & Berruti A 2022 Supportive therapies in patients with advanced adrenocortical carcinoma submitted to standard EDP-M regimen. Endocrine 77 438–443. (10.1007/s12020-022-03075-y) 35567656 PMC9385801

[bib30] Vaksman O Hetland TE Trope’ CG Reich R & Davidson B 2012 Argonaute, Dicer, and Drosha are up-regulated along tumor progression in serous ovarian carcinoma. Human Pathology 43 2062–2069. (10.1016/j.humpath.2012.02.016) 22647351

[bib31] Yan M Huang H-Y Wang T Wan Y Cui S-D Liu Z-Z & Fan Q-X 2012 Dysregulated expression of dicer and drosha in breast cancer. Pathology Oncology Research 18 343–348. (10.1007/s12253-011-9450-3) 21898071

[bib32] Ye Z Jin H & Qian Q 2015 Argonaute 2: a novel Rising Star in cancer research. Journal of Cancer 6 877–882. (10.7150/jca.11735) 26284139 PMC4532985

[bib33] Yi R Qin Y Macara IG & Cullen BR 2003 Exportin-5 mediates the nuclear export of pre-microRNAs and short hairpin RNAs. Genes and Development 17 3011–3016. (10.1101/gad.1158803) 14681208 PMC305252

[bib34] Yoshida K Yokoi A Yamamoto Y & Kajiyama H 2021 ChrXq27.3 miRNA cluster functions in cancer development. Journal of Experimental and Clinical Cancer Research 40 112. (10.1186/s13046-021-01910-0) 33766100 PMC7992321

[bib35] Zheng S Cherniack AD Dewal N Moffitt RA Danilova L Murray BA Lerario AM Else T Knijnenburg TA Ciriello G, *et al.* 2016 Comprehensive pan-genomic characterization of adrenocortical carcinoma. Cancer Cell 29 723–736. (10.1016/j.ccell.2016.04.002) 27165744 PMC4864952

