# Supplementary material for: AGO2 protein: a key enzyme in the miRNA pathway as a novel biomarker in adrenocortical carcinoma
Source: Endocr Relat Cancer. 2024 Nov 20;31(12):e240061. doi: 10.1530/ERC-24-0061 (PMC11623120; doi:10.1530/ERC-24-0061)
Supplement: Supplementary Figure 1A:Co-expression of AGO2 with prognostically significant miRNAs in ACC The top four miRNAs highly expressed in the COC3 group-TCGA-ACC, which is associated with poor prognosis (hsa-miR-183-5p, hsa-miR-139-3p, hsa-miR-182-5p, and hsa-miR-196a-5p), demonstrated a positive correlat [file supplementary_figure_1.pdf]

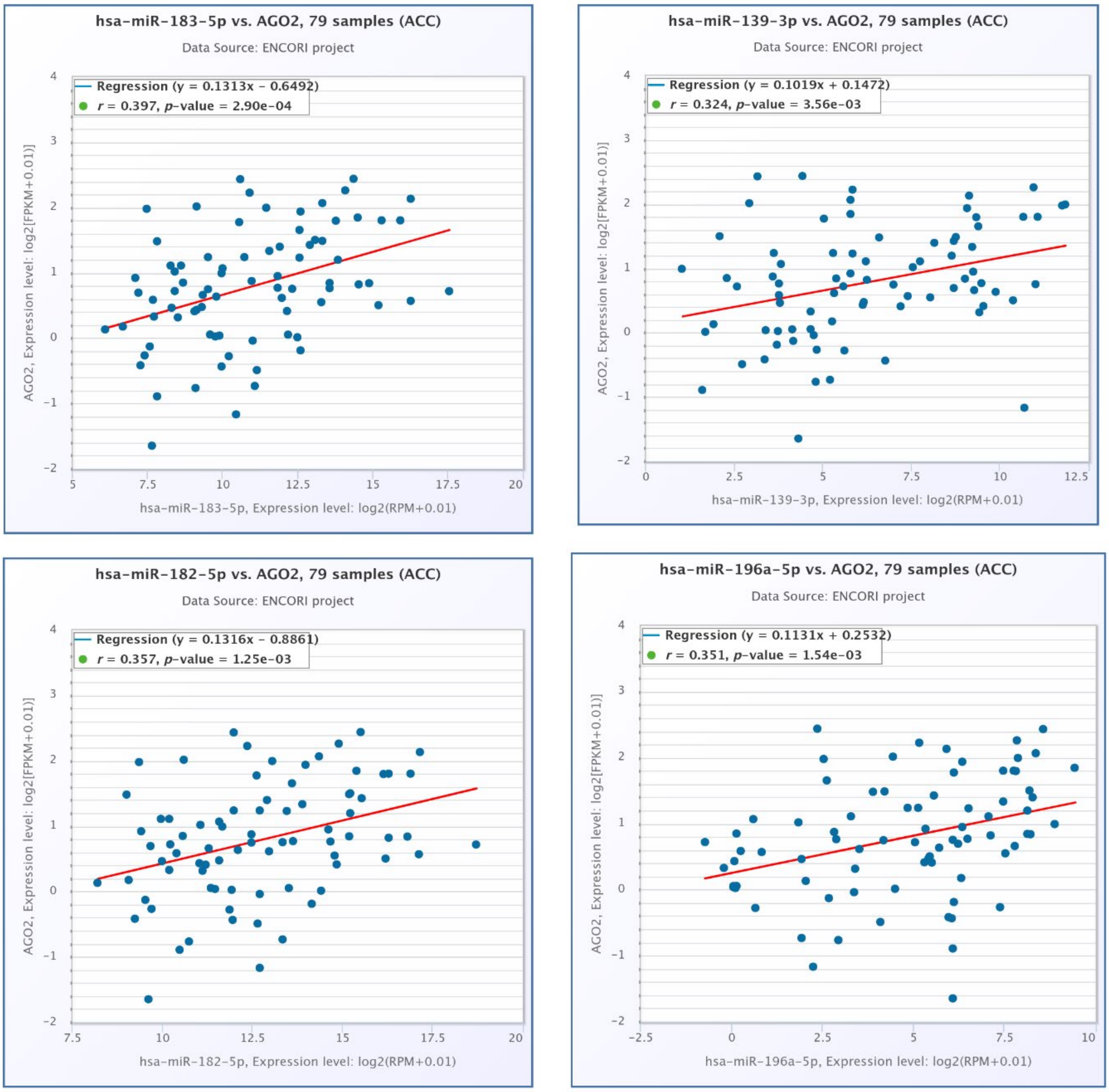

Supplementary Figure 1A:Co-expression of AGO2 with prognostically significant miRNAs in ACC The top four miRNAs highly expressed in the COC3 group-TCGA-ACC, which is associated with poor prognosis (hsa-miR-183-5p, hsa-miR-139-3p, hsa-miR-182-5p, and hsa-miR-196a-5p), demonstrated a positive correlation with AGO2 expression.

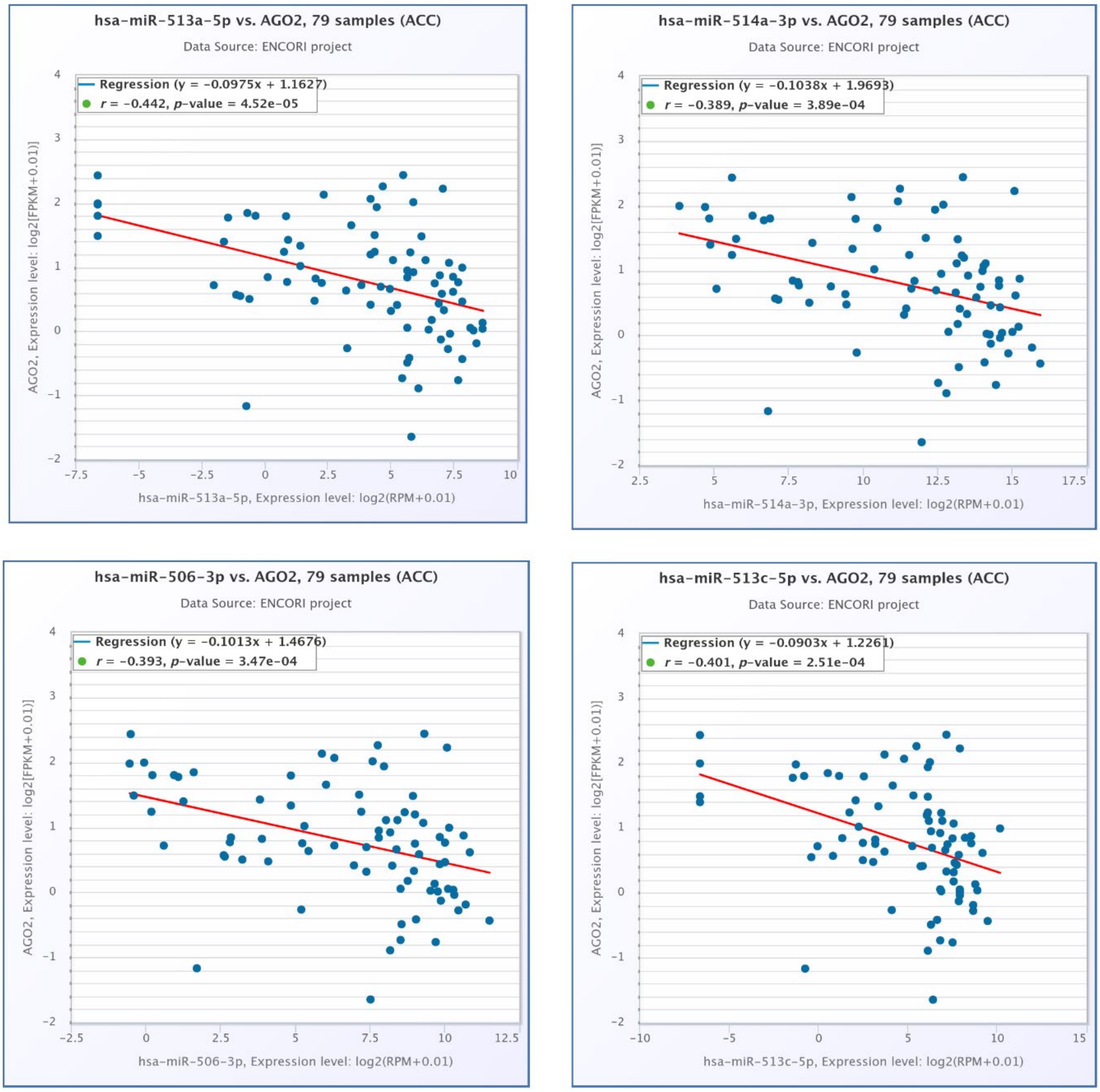

Supplementary Figure 1b AGO2 showed negative correlation with the top four upregulated miRNAs linked to the TCGA-ACC COC1 cluster with better prognosis (hsa-miR-513a-5p, hsa-miR-514a-3p, hsa-miR-506-3p, hsa-miR-513c-5p).
