# Supplementary material for: AGO2 protein: a key enzyme in the miRNA pathway as a novel biomarker in adrenocortical carcinoma
Source: Endocr Relat Cancer. 2024 Nov 20;31(12):e240061. doi: 10.1530/ERC-24-0061 (PMC11623120; doi:10.1530/ERC-24-0061)
Supplement: Supplementary Table 1: Pan-Cancer AGO2 expression and survival analysis in TCGA cohorts. This table summarizes the hazard ratios (HR) for AGO2 expression across 32 TCGA cancer types, highlighting its prognostic significance, particularly in ACC with an HR of 7.07 (p value 2.80E-06) [file supplementary_table_1.pdf]

Supplementary Table 1: Table 1. **Pan-Cancer AGO2 expression and survival analysis in TCGA cohorts**. This table summarizes the hazard ratios (HR) for AGO2 expression across 32 TCGA cancer types, highlighting its prognostic significance, particularly in ACC with an HR of 7.07 (p value 2.80E-06)

Cancer	Cancer Number	p-value (significant thershold <0.05)	HR
ACC	79	2.80E-06	7.07
MESO	85	0.00053	2.36
UCEC	537	0.0052	1.83
SARC	261	0.0092	1.71
KIRP	288	0.016	2.15
CHOL	36	0.044	0.38
LGG	523	0.065	1.39
BRCA	1082	0.087	1.32
THYM	118	0.1	0.29
LIHC	369	0.12	1.32
KICH	64	0.21	2.35
UVM	80	0.23	1.69
CESC	306	0.27	1.3
OV	374	0.36	1.13
READ	159	0.36	0.69
LUAD	503	0.43	1.13
UCS	56	0.43	1.32
ESCA	162	0.49	0.84
STAD	365	0.5	0.89
HNSC	495	0.56	0.92
PCPG	183	0.57	1.52
BLCA	406	0.61	1.08
LUSC	469	0.67	1.06
SKCM	440	0.73	0.95
TGCT	139	0.75	0.72
PRAD	495	0.76	1.22
LAML	75	0.79	0.93
PAAD	178	0.8	1.06
KIRC	517	0.81	1.04
DLBC	47	0.82	1.18
THCA	509	0.84	1.11
COAD	447	0.87	1.03
