# Supplementary material for: AGO2 protein: a key enzyme in the miRNA pathway as a novel biomarker in adrenocortical carcinoma
Source: Endocr Relat Cancer. 2024 Nov 20;31(12):e240061. doi: 10.1530/ERC-24-0061 (PMC11623120; doi:10.1530/ERC-24-0061)
Supplement: Supplementary Table 2: Comparative Analysis of AGO2 mRNA and Protein Levels Against Clinicopathological Parameters and Their Prognostic Significance in TCGA-ACC and an Independent ACC Cohort. [file supplementary_table_2.pdf]

**Supplementary Table 2: Comparative Analysis of AGO2 mRNA and Protein Levels Against Clinicopathological Parameters and Their Prognostic Significance in TCGA-ACC and an Independent ACC Cohort.**

| Parameters | TCGA-ACC cohort (n=79) | AGO2 mRNA expression in TCGA (log2) | p-value | Collected-ACC cohort (n=15) (Kollings Tumour bank) | AGO2 Protein concentration in collected cohort (ng/ml) | p-value |
|---|---|---|---|---|---|---|
| | | | | | | |
| **Diagnosis Age** | 46.7 ± 15.77 | | 0.672 | 46.27 ± 15.47 | | 0.833 |
| | | | | | | |
| <49 | 39 | 6.64 ± 0.77 | | 9 | 6.66 ± 2.71 | |
| >49 | 40 | 6.72 ± 0.9 | | 6 | 7.03 ± 4.14 | |
| | | | | | | |
| **Sex** | | | 0.254 | | | 0.484 |
| Female | 48 | 6.59 ± 0.8 | | 8 | 6.24 ± 2.97 | |
| Male | 31 | 6.82 ± 0.88 | | 7 | 7.46 ± 3.6 | |
| | | | | | | |
| **Overall survival status** | | | <0.001 | | | 0.009 |
| | | | | | | |
| Alive | 51 | 6.44 ± 0.7 | | 9 | 5.16 ± 2.42 | |
| Deceased | 28 | 7.11 ± 0.89 | | 6 | 9.28 ± 2.72 | |
| | | | | | | |
| **Weiss score** | | | 0.003 | | | 0.008 |
| | | | | | | |
| Weiss score (2-5) | 30 | 6.32 ± 0.8 | | 4 | 3.43 ± 1.86 | |
| Weiss score (6-9) | 31 | 6.9 ± 0.83 | | 11 | 8.04 ± 2.69 | |
| | | | | | | |
| **Pathologic stage** | | | 0.011 | | | 0.004 |
| | | | | | | |
| Stage I-II | 46 | 6.49 ± 0.78 | | 5 | 3.76 ± 1.77 | |
| Stage III-IV | 31 | 6.93 ± 0.87 | | 10 | 8.33 ± 2.64 | |
| | | | | | | |
| **Cluster of clusters (CoCs)** | n=76 | | 0.036 (COC1 vs COC3) | | | |
| COC1 (Disease progression rate: (7%) | 33 | -0.16 ± 0.91 | | | | |
| COC2 (Disease progression rate: (56%) | 19 | -0.18 ± 1.14 | | | | |
| COC3 (Disease progression rate: (96%) | 24 | 0.31 ± 1.01 | | | | |

The data are presented as the means ± SDs or n (numbers).
Abbreviations: TCGA, The Cancer Genome Atlas
